# Identifying and addressing the anthropogenic drivers of global change in the North Sea: a systematic map

**DOI:** 10.1186/s13750-025-00377-2

**Published:** 2025-12-02

**Authors:** Alexandra M. Blöcker, Dominik Auch, Helene M. Gutte, Johanna Biederbick, Rémy Asselot, Leonie Färber, Gregor Börner, Elvis Kamberi, Frane Madiraca, Claudia Ofelio, Laurin Steidle, Fabien Moullec

**Affiliations:** 1 Institute for Marine Ecosystem and Fisheries Science (IMF), Center for Earth System Research and Sustainability (CEN), University of Hamburg, 22767 Hamburg, Germany; 2 Institute of Carbon Cycles, Helmholtz-Centre Hereon, Max-Planck-Strasse 1, 21502 Geesthacht, Germany; 3 BRGM, French Geological Survey, 24 Avenue Léonard de Vinci, 33600 Pessac, France; 4 GEOMAR Helmholtz Centre for Ocean Research Kiel, Wischhofstr. 1-3 Geb. 4/325, 24148 Kiel, Germany; 5 Department of Aquaculture and Fisheries, Agricultural University of Tirana, St. Paisi Vodica 1025, Tirana, Albania; 6 Valencian International University, C/ Pintor Sorolla 21, 46002 Valencia, Spain; 7 MARBEC, Univ Montpellier, CNRS, Ifremer, IRD, Montpellier, France

**Keywords:** Climate change, Direct exploitation, Pollution, Biological invasions, Sea-use change, Anthropocene, Marine ecosystem, Research gap, Research trend, Evidence-based

## Abstract

**Background:**

Marine ecosystems worldwide face extreme stress from human activities, with the North Sea being particularly affected and experiencing altered processes. To assess anthropogenic drivers for sustainable management, the Millenium Ecosystem Assessment (MEA) and the Intergovernmental Science-Policy Platform on Biodiversity and Ecosystem Services (IPBES) distinguished five main anthropogenic drivers: direct exploitation of fish and seafood, sea use change, human-driven climate change, pollution, and invasive alien species. However, evidence of the drivers’ relevance and their potential effects on species and the environment over time remains scarce. This systematic map provides knowledge on the five main anthropogenic drivers in the North Sea from 1945 to 2020 and identifies potential knowledge gaps in terms of management implications.

**Methods:**

To identify relevant articles we used our published systematic map protocol. We conducted systematic searches of academic and grey literature in English, German, and French in online databases (Web of Science, Scopus, PubMed, AquaDocs). The search followed a Population-Exposure-Comparison-Outcome framework and included the period January 1945 to December 2020. A total of 22,511 articles were deduplicated and screened by title and abstract, the remaining 5795 were screened full-text to provide a widely integrated evidence base. A set of 3356 articles were retained following eligibility criteria and were included in the final database. We extracted information on drivers in detail and their effects on study populations within different areas in the North Sea. Knowledge clusters and gaps were identified from the scientific effort and are synthesized narratively.

**Results:**

Out of the 3356 articles, the majority focused on pollution throughout the entire period of 75 years. Research interest has increased in climate change and biological invasion only in the most recent decades. We identified knowledge clusters in the southern North Sea, especially in ICES standard species areas 6 and 7, which has the most articles overall, mainly emphasizing pollution. Northern areas were in contrast studied the least. The effects of pollution were mainly linked to changes in chemical water properties and to contamination levels for benthos and fish. The other drivers were rather associated with changes in biomass or abundance, with a strong focus on fish and benthos populations. A key knowledge gap was on the effects of global change, herein defined as simultaneous assessment of all five drivers, at different organizational levels and therein on different populations.

**Conclusions:**

This systematic map reveals substantial peer-reviewed evidence on the five main anthropogenic drivers in the North Sea. The map uncovers a strong increase in research interest regarding these drivers over the years, with a strong focus towards pollution and southern North Sea areas. Despite the increasing importance of climate change effects, this map highlights limited research effort on it. As ecosystem management nowadays strives for sustainable use of marine systems, it is more important than ever to understand linkages between drivers, potential cumulative effects and possible repercussions. The map revealed a strong knowledge gap regarding these linkages due to global change. On this basis, further systematic reviews can acknowledge these gaps, identifying the drivers’ impacts and their quick evolvement to support management decision-making at various governance levels.

**Supplementary Information:**

The online version contains supplementary material available at 10.1186/s13750-025-00377-2.

## Background

Marine ecosystems worldwide are heavily impacted and largely altered by human activities [1–4]. Continuously increasing anthropogenic pressures, especially due to demographic pressures in coastal areas, cause direct (e.g. fishing exploitation) and indirect (e.g. bioaccumulation) effects specifically on the oceans’ functionality, resilience and biodiversity leading to severe repercussions in human well-being and ecosystem services [4–8]. In the last decades, close to 66% of the ocean’s surface has experienced cumulative impacts, for example due to invasive species or nutrient enrichment [1, 9–11].

For marine systems, the Millennium Ecosystem Assessment (MEA) [12] and the Intergovernmental Science-Policy Platform on Biodiversity and Ecosystem Services (IPBES) [13] identified five main anthropogenic drivers, which directly affect ecosystem processes: direct exploitation of fish and seafood, sea use change, human-driven climate change, pollution, and invasive alien species. Global change is considered to include the impacts of all these five drivers at once. Whereas direct exploitation was considered as the prime driver affecting marine ecosystems and biodiversity for several decades, the latter four are considered as recently increasing threats with the possibility to be the major drivers leading to biodiversity loss in the future [13–15]. Given these rapid changes in anthropogenic drivers, marine ecosystems are already severely impacted and are expected to be further affected in the near future [16–20].

Among all oceans worldwide, the North Sea is a paradigm for being one of the most human-influenced marine ecosystems in the Northeast Atlantic Ocean [10, 21]. The North Sea experiences strong effects of multiple anthropogenic pressures with increasing tendency given about 184 million inhabitants surrounding the sea and over 500 inhabitants per km^2^ in coastal areas [22, 23]. The European Green Deal implying EU’s climate neutrality by 2050, for instance, strengthens the construction of offshore renewable energy in the North Sea (300 GW by 2050). The transition of vast North Sea areas into “a green power plant” is setting a new dimension of industrial sea use change [24, 25]. However, specifically climate change is predicted to be the key player in the future, outpacing the other drivers [13, 16, 26, 27]. As the North Sea is considered already as a hot spot of climate change due to strong water temperature increases [28], severe impacts can be expected on its ecosystem functioning and biodiversity [29, 30].

Due to the increasing pressure of the five drivers on marine ecosystems, there is an urgent need to achieve the Sustainable Development Goal (SDG 14) established by the United Nations (conserve and sustainably use oceans, seas and marine resources for sustainable development) [31]. Additionally, there is a need to reach the new biodiversity goals and targets of the post-2020 global biodiversity framework [15], and to progress towards an effective science-based and integrated ocean management system [32]. Understanding how anthropogenic drivers affect marine ecosystems in space and time can promote the achievement of these goals and support the maintenance and restoration of key ecosystem functions [33, 34]. Also, the ongoing UN Decade of Ocean Science for Sustainable Development 2021–2030 and its 2030 Agenda can be supported by a retrospective evaluation of climate change by clearly identifying knowledge gaps and clusters of the main anthropogenic drivers in marine research [35–38]. Along with their distinct relevance in impacting marine ecosystems, the five main anthropogenic drivers appear to be studied unequally. As an example, climate change is globally the most studied driver of biodiversity loss in the last decade, whereas studies on pollution and habitat change were rather rare [39].

Due to extensive monitoring by several countries organized in the International Council for the Exploration of the Sea (ICES) and the Convention for the Protection of the Marine Environment of the North-East Atlantic (OSPAR), the North Sea is a well-studied and data-rich system [28, 40, 41]. Despite this robust scientific foundation, only few studies analyze or synthesize research on global change, and how this research is connected and correlated with the magnitude and trends of the five anthropogenic drivers.

Here, we present a systematic map that focuses on academic and grey literature and highlights the effects of the five main anthropogenic drivers on the North Sea ecosystem. Our study aims to examine and provide an overview of the scientific research on these drivers across various study populations and geographical areas, in order to identify knowledge gaps and to connect scientific knowledge generated over the last 75 years (1945–2020). Following our previously published systematic map protocol [42], the present systematic map shows the state of scientific research on the five anthropogenic drivers, and provides an asset towards reaching the UN Decade of Ocean Science for Sustainable Development.

## Objective of the review

The main objective of the systematic map is to give a thorough overview of the knowledge and methods used to assess the impacts of the five main drivers (direct exploitation of fish and seafood, sea use change, human-driven climate change, pollution, and invasive alien species) on the marine ecosystem in the North Sea since the 1940s by focusing on the ecological, physical and chemical components using an ecosystem approach. In addition to these five drivers, global change was included as another category, representing the existence of all five main drivers simultaneously. Socio-economic impacts and indirect anthropogenic drivers (e.g., governance, economic, demographic drivers) were out of scope. A detailed description of the objectives is available in our protocol [42].

The primary overarching research question this study addresses is:

How did the research interest in anthropogenic drivers of marine ecosystem changes develop over the past 75 years (1945–2020) regarding the North Sea?

This research question was subsequently complemented by the following set of secondary research questions:

1. *Which direct drivers have been neglected by “modern” (since 1940’s) marine science? (Knowledge gaps)*
2. *What is the proportion of research dedicated towards testing responses to observed or anticipated impacts?*
3. *Facing global change, which changes in nature’s contribution to people are least studied?*
4. *Is there a relationship among advances in applied methodologies, conceptual frameworks and acknowledged gaps in knowledge?*
5. *Are ecological shifts in the North Sea reflected in funded research programs and topics?*
6. *What are the main future avenues of research in marine science?*

To answer these questions, we created a systematic map, using the following components:

- Population: any biotic and abiotic component of the North Sea marine ecosystem, e.g. plant and animal species (excluding humans) and e.g. seawater and sediment properties, respectively. Geographical scope: ICES divisions IVa, IVb and IVc; ICES standard species areas 1–7 [43, 44], Norwegian coast.
- Exposure(s)/intervention(s): the five anthropogenic drivers (direct exploitation of fish and seafood, sea use change, human-driven climate change, pollution, and invasive alien species) will be considered as the exposures. Also, global change representing the impacts of all five main drivers at the same time is considered as an holistic exposure. Hence, studies that include all five drivers simultaneously will be labeled as global change, instead of labeling all five drivers separately. Management, conservation and protection, and restoration will be considered as interventions.
- Comparator: studies will not be required stricto sensu to have a comparator.
- Outcome(s): changes in the five anthropogenic drivers and their relation to populations studied (No outcomes were predefined, i.e., all possible outcomes).

## Methods

This systematic map was performed in accordance with the previously published protocol [42] and followed as precisely as possible the Collaboration for Environmental Evidence (CEE) Guidelines and Standards for Evidence Synthesis in Environmental Management [45]. The map complies with the RepOrting standards for Systematic Evidence Syntheses (ROSES) [46] (Additional file 1). Updates in the process of study screening and coding are listed in the following section.

### Deviations from the protocol

The systematic map was conducted following the methods described in the published protocol [42], including deviations described as followed: in contrast to the protocol, information to answer secondary questions 2 was not retrieved consistently. The label was located last in the overview, which might be a reason for overlooking answering the solution investigated (not tested, management, conservation and protection, restoration). The responses given are not sufficient to provide a good and comprehensive answer for secondary question 2. Also, information about funding was not extracted during the coding stage as the funding information gained was little during the test periods, i.e., excluding information to answer secondary question 5. Metadata on the countries and lab of authors (bibliographic information) was not extracted in the end, as the original idea to link publications to authors’ countries and funding was not conducted. Also, the location of the authors is not always representative for where the studies were carried out. It became clear that information on where the study itself was spatially performed, was rather of high relevance. Moreover, information regarding the air compartment (e.g., aerosols) was not included in the data extraction process, as the focus was strictly on the marine compartment. In addition, the label global change was added to the exposures’ list. Global change was considered if a study included all five main drivers simultaneously, and therefore represents these as a holistic label. The choice to include this label in this systematic map was made to highlight how much literature takes an integrated approach by including all five main drivers. In this way, knowledge clusters and gaps regarding simultaneous effects on study population could be identified. Moreover, the contributing authors of this systematic map differed from the authors of the previously published protocol. This deviation was due to changes in authors’ affiliations and time constraints when the systematic map phase started. To manage the screening of more than 5795 full text documents, newly involved authors (HMG, JB, EK, FMa) were trained along with previously involved authors (AMB, DA, RA, LF, CO, LS, FMo) until the Fleiss’ kappa (K) parameter reached > 0.6. Due to the slightly reduced number of reviewers and the large number of studies included (5795 in total), the conflict solving process was conducted by four reviewers instead of two. The change of group composition also led to the exclusion of reviewing literature in Spanish and Italian, due to the lack of a second reviewer in these languages.

### Search for articles

The search terms were based on the major anthropogenic drivers listed in the global assessment report of the IPBES [13]: direct exploitation of fish and seafood, sea use change, human-driven climate change, pollution, and invasive alien species. All drivers’ synonyms were discussed and search terms were tested repeatedly for comprehensiveness in Web of Science (WoS) by comparing their ability to find a list of benchmark articles. In order to improve comprehensiveness, wildcards (*) were used where word endings potentially vary. NEAR/3 was used as a proximity operator to find records where the order of words was slightly modified. Besides the term ‘North Sea’, regional terms such as ‘Buchan Deep’ or ‘Wadden Sea’ were added to increase comprehensiveness. In WoS, search terms were inserted in the field code “Topic” corresponding to an automatic algorithm-based search for frequently used terms in the reference list. This method covers a search in title, abstract, publications’ keywords and “keyword plus”, an automatic algorithm-based search for frequently used terms in the reference list. More than 95% of articles from the benchmark list were found indicating a high coverage of available literature. The search in WoS, Scopus and PubMed required minor changes. Thus, ‘TS’ (WoS) was replaced by ‘Title-ABS-KEY’ in Scopus and by ‘[Title/Abstract]’ in PubMed. The final combination excluding regional differentiation was the following:

#### Exposure terms

TS = ("climat*" NEAR/3 "chang*" OR "global warming" OR "carbon dioxide" OR "CO2" OR "acidification" OR "deoxygen*" OR "oxygen" NEAR/3 "loss" OR "decreas*" NEAR/3 "oxygen" OR "reduc*" NEAR/3 "oxygen" OR "sea surface temperature*" OR "marine warming" OR "climate warming" OR "ocean warming" OR "temperature*" NEAR/3 "chang*" OR "seawater warming" OR "sea water warming" OR "rising temperature*" OR "greenhouse" OR "green house" OR "invasi*" OR "alien" OR "introduc*" NEAR/3 "species" OR "non-native" OR "nonnative" OR "endangered" OR "biodiversity" OR "biological diversity" OR "unsustainabl*" OR "abundance" NEAR/3 "change*" OR "distribution" NEAR/3 "change*" OR "habitat*" NEAR/3 "chang*" OR "habitat*" NEAR/3 "loss" OR "fragmentation" OR "habitat*" NEAR/3 "qualit*" OR "habitat*" NEAR/3 "fragment*" OR "habitat*" NEAR/3 "modif*" OR "habitat*" NEAR/3 "degrad*" OR "habitat*" NEAR/3 "decline" OR "habitat*" NEAR/3 "destabili*" OR "habitat*" NEAR/3 "destruction" OR "habitat*" NEAR/3 "destroy*" OR "ecosystem*" NEAR/3 "degrad*" OR "ecosystem*" NEAR/3 "destruction" OR "ecosystem*" NEAR/3 "decline*" OR "ecosystem*" NEAR/3 "qualit*" OR "ecosystem*" NEAR/3 "fragment*" OR "ecosystem*" NEAR/3 "chang*" OR "landscape" NEAR/3 "chang*" OR "sea-use chang*" OR "offshore" NEAR/3 "wind farm*" OR "offshore" NEAR/3 "wind park*" OR "OWF*" OR "overfish*" OR "overexploit*" OR "overharvest*" OR "overhunt*" OR "over hunt*" OR "over fsh*" OR "over exploit*" OR "over harvest*" OR "exploit*" OR "fishing" OR "fisheries" OR "fishery" OR "pollut*" OR "eutrophicat*" OR "trophic amplifidegrecation" OR "noise*" NEAR/3 "impact*" OR "noise*" NEAR/3 "increas*" OR "noise level*" OR "light level*" OR "light" NEAR/3 "impact*" OR "light" NEAR/3 "increas*" OR "nitrogen*" NEAR/3 "increas*" OR "nitrogen*" NEAR/3 "impact*" OR "nutrient*" NEAR/3 "loading" OR "ecotoxic*" OR "environment*" NEAR/3 "toxic*" OR "ecologic*" NEAR/3 "toxic*" OR "environment*" NEAR/3 "toxic*" OR "heavy metal*" OR "oil spill*" OR "oil leak*" OR "runof" OR "run of" OR "microplastic*" OR "micro plastic*" OR "contamina*" OR "water qualit*" OR "cruise ship*" OR "trawl*" OR "dredging" OR "drilling" OR "dumping" OR "deglaciat*" OR "anthropogenic*" OR "human induced" OR "human driven" OR "human stressor*" OR "human pressure*" OR "human* impact*").

#### Geographical scope

AND TS = ("North Sea" OR "Brent Group" OR "Sleipner Vest Field" OR "Sleipner Field" OR "Maar Bank" OR "Pobie Bank" OR "Forty Mile Ground" OR "Viking Bank" OR "Viking Graben" OR "Little Halibut Bank" OR "Smith Bank" OR "Moray Firth" OR "Buchan Deep" OR "Fladen Ground" OR "Utsira high" OR "Utsira Formation" OR "Ling Bank" OR "Revet" OR "Norwegian Trench" OR "Eigersunds Bank" OR "Little Fisher Bank" OR "Great Fisher Bank" OR "Fisher Banks" OR "Jutland Bank" OR "Jutland Coastal Current" OR "Horns Rev" OR "Long Forties" OR "Scalp Bank" OR "Devil’s Hole" OR "Firth of Forth" OR "Farn Deeps" OR "Dogger Bank" OR "Silverpit Crater" OR "Outer Silver Pit" OR "Inner SilverPit" OR "Silver Pit" OR "Norfolk Banks" OR "The Wash" OR "Southern Bight" OR "Broad Fourteens" OR "Frisian Front" OR "Cleaver Bank" OR "Oyster Ground" OR "German Bight" OR "German Bight Water" OR "Heligoland Bight" OR "Wadden Sea" OR "Strait of Dover" OR "Central Graben" OR "Snorre Field" OR "Wee Bankie" OR "North Sea Canal" OR "Fair Isle Current" OR "Norwegian Trench" OR "Hild Field" OR "Tern Field" OR "Middelkerke Bank" OR "Fulmar Formation" OR "Ninian Field" OR "Frigg Field" OR "Statfjord Field" OR "Statfjord Formation" OR "Gullfaks Field" OR "Pomeranian Bight" OR “Norwegian Deep" OR "Broad Fourteens Basin" OR "North Sea Flemish Banks" OR "Norwegian Channel" OR "Ekofisk Field" OR "North West Hutton Field" OR "Leman Field" OR "Aberdeen Bank" OR "Plaice Box" OR "Troll Field" OR "Mellum I.").

### Search limitations and sources

The search was conducted for studies published from January 1945 to December 2020 and no further updates were made due to the limited number of researchers involved (lack of manpower) and the great number of articles already reviewed. The search term focused on English studies. Studies published in French and German language were nonetheless reviewed by those reviewers being fluent in the respective language. Studies in other languages were excluded. The limitation to these languages is expected to be of minor importance as English is largely accepted as the scientific language in coastal states of the North Sea. In addition, the ultimate combination of search terms encompasses the five main anthropogenic drivers and several geographic areas. However, it might have missed other potential emerging threats in the North Sea in specific areas that are not covered.

Published literature was searched using Web of Science Core Collection on the Web of Science platform (Clarivate), Scopus (Elsevier) PubMed (https://pubmed.ncbi.nlm.nih.gov/) and AquaDocs (see Additional File 2 for all search results). Additional grey literature was extracted via BASE (Bielefeld Academic Search Engine; https://www.base-search.net/) and CORE (https://core.ac.uk/) extracting the first 300 results over the entire period of interest, following Haddaway et al. [46]. Additionally, 300 results per driver and decade were extracted from Google Scholar (https://scholar.google.com/) using simplified search terms per individual anthropogenic driver and the ‘Advanced Search’ option. Furthermore, organizational websites were searched for one of the five major direct anthropogenic drivers (“climate change”; “overexploitation”; “invasive species “; “habitat change”, “pollution”) in conjunction with the term “North Sea”. The literature search was completed by references cited in the benchmark articles. Literature from all searches was compiled in SysRev, an online platform for collaborative document reviews and automated data extraction (https://sysrev.com/ [47]). Duplicates coming from different platforms were removed.

### Article screening and study eligibility criteria

#### Screening process

The screening was conducted in two consecutive steps, starting with a title and abstract screening and a subsequent full-text screening. While the first screening was conducted by six reviewers, 10 reviewers (the authors) were involved in the full text screening process. Prior to both screenings multiple training sessions were performed, discussing and clarifying all issues of disagreement in the group. A detailed coding guide was prepared and used consistently. The start of both screening stages was determined after reaching a good level of consistency concerning inclusion/exclusion decisions, using the Fleiss’ kappa (K) parameter > 0.6. To ensure impartiality, reviewers skipped any studies which they were authoring themselves. During both screening steps, each study was screened by two reviewers. During the phase of title and abstract screening, decisions were made conservatively, meaning that studies were included at this step when being in doubt. Two additional reviewers screened all studies with conflicting screening results. During the full text screening the same process was conducted, including two reviewers per study and four reviewers for papers with conflicting coding results.

### Eligibility criteria

Articles found with the search strategy had to meet a set of eligibility criteria described in our protocol [42]:

Relevant spatial and temporal focus: literature was included when it included ICES subdivisions IVa, IVb and/or IVc (ICES standard species areas 1–7, Norwegian coast [43, 44]), including fjords, estuaries and temporarily flooded areas such as marshlands or beaches. Literature with a broader focus (Northeast Atlantic, global perspective) had to explicitly include the North Sea. Studies from adjacent areas alone (English Channel, Kattegat, Skagerrak) were excluded from the analysis. Only literature dealing with anthropogenic drivers in the period from January 1945 to December 2020 was considered as relevant.

Relevant populations: studies with a focus on anthropogenic effects on the abiotic (chemical, physical) or biotic components (plants, animals) of the North Sea (water column, sediment/substrate) including temporarily relevant components such as sea birds were included. Effects on humans were considered irrelevant. Literature solely investigating atmospheric effects was excluded likewise.

Relevant exposure: literature was required to explicitly focus on the effect of one of the five major anthropogenic impacts (direct exploitation of fish and seafood, sea use change, human-driven climate change, pollution, and invasive alien species). Literature dealing with respective management/mitigation actions was included if analyzing their effects. Socio-economic or political papers were, on the contrary, excluded if they were not explicitly linked to anthropogenic effects on the North Sea ecosystem. Literature on climate variability that was not explicitly linked to climate change was excluded as well. Management actions related to climate change mitigation, such as engineering solutions for carbon capture and storage (CCS) were considered as irrelevant if lacking any analysis of direct impacts on the North Sea ecosystem (e.g., leakage of CO_2_ from a storage site).

Relevant study design: a broad variety of studies was included, covering primary research articles, book chapters, reviews, conference papers, meta-analyses, reports, theses, and others to assess potential changes in how anthropogenic effect analyses are approached. Therefore, our dataset covers a broad methodological variety analyzing historic, current, and future impacts of anthropogenic drivers. Methodological papers without an application of these methods to the North Sea ecosystem were considered as irrelevant. Scientific studies where only an abstract was found were removed.

### Study validity assessment

A validity assessment was not conducted. The huge variety of literature types and the wide temporal period of publication provide information on study design and approach, which will facilitate an eventual quality assessment for specific research questions later on.

### Data coding strategy

During the data coding step, a comprehensive set of meta-data was extracted including bibliographic information, information concerning the drivers on two detail levels, insights into the effect assessed, the methodological approach (Table 1), and the analyzed impacts (Table 2, see Additional file 3 for all labels and their explanations). Bibliographic information on authors were only obtained for author names and therefore number of authors too. Countries and labs of authors were no included during the extraction process, as they were meant to be linked to funding information (not retrievable). If a study analyzed more than three impacts simultaneously, the label ‘multiple impacts’ was applied. The labels ‘not specified’ for ICES locations and ‘not applicable’ for organizational level (e.g., individual or community) were used to indicate that no specific area in the North Sea was mentioned in the article (e.g., only North-east Atlantic) and none living organizational level applied (e.g., chemical or metal substances). SysRev was used as the platform for coding. Before starting the coding, 12 rounds of training were conducted during which categories and their labels were discussed, and thresholds defined. During the coding process, each study was reviewed twice and in case of conflicts a third person carried out the conflict solving decision.

**Table 1:** Categories used for coding studies

Category	Type of data
Bibliographic information	Author names Numbers of authors Publication type Publication source Publication year Number of citations Current impact factor of the journal
Subject of investigation	Type of driver and precision of driver Analyzed impact (Table 2) Affected population (incl. biotic and abiotic pop.) Organizational level (e.g., species, population) ICES major (IVa-c) and medium (ICES standard species areas 1–7, Norwegian coast) areas Habitat type
Methodological approach	Main methodology Spatial and temporal dimension Solution investigated

**Table 2:** Impacts extracted from coding studies

Analyzed impacts	Comment
Change in biomass/abundance	Abundance/Density/includes catch volumes as well
Change in demographic structure	Age/size/sex ratio etc. size spectrum
Change in life history traits	Number of generations, rates of reproduction, mortality/survival/development, age and size at maturity, size at birth, longevity
Change in spatial distribution	Latitudinal, longitudinal, vertical
Degradation of habitat	Degradation/destruction of a habitat
Change in diversity	Species richness, other biodiversity indicators (e.g., functional diversity)
Change in physiology	Metabolic processes, energetics, stress, regulation processes, sexual inversion
Change in contamination level	Pollutant concentration in (organs/tissues of) living organisms
Change in phenology	Timing of seasonal activities (bloom formation, breeding, migration etc.)
Change in genetic	Changes in genes/alleles
Bioaccumulation in food web	Gradual accumulation of substances through trophic interactions
Change in trophic functioning	Change in trophic levels, food web structures (e.g., bottom-up/top-down), trophic efficiency, predation/grazing pressures
Change in biochemical fluxes	Phosphorus, Carbon, Nitrogen, ratios (e.g. Cd:Ca,Cd:PO4)
Change in harmful algal blooms	Increase/decrease of harmful algal blooms
Change in extreme events	Marine heat waves, storm surges, floods, etc
Change in physical water properties	Stratification, currents, warming
Change in physical sediment properties	Grain density, grain size structure, porosity, seafloor structure, geomorphological changes
Change in chemical water properties	pH, salinity, oxygen concentration, concentration of pollutants
Change in chemical sediment properties	pH, salinity, oxygen concentrations, concentration of pollutants; includes particulate organic matter in the water column
Coastal in erosion and sedimentation	Geomorphological changes of coastlines
Change in toxins	Biological toxins (e.g., Paralytic shellfish poisoning)
Quantification of the driver	If the driver was only quantified and assessed (e.g., fishing effort, plastic litter)

### Data mapping method

The detected evidence base was compiled into a Microsoft Excel database, which was explored and cleaned for duplicates using the package tidyverse [48, 49] in R [50, 51]. Plots, Sankey diagrams, and maps were created using the R packages ggplot [7, 52], network3D [53, 54] and mapplots [55], respectively. To highlight knowledge gaps and clusters, we summarized the data accordingly to the five main anthropogenic drivers, ICES standard species areas, precision of drivers (the driver in detail), analyzed impacts (Table 2), nature of the study population, organizational level (individual, population, community and ecosystem level), and the main methodology used. Based on the outcomes, we show how research regarding the five main anthropogenic drivers (direct exploitation of fish and seafood, sea use change, human-driven climate change, pollution, and invasive alien species) in the North Sea has changed over 75 years, and make suggestions on the importance of future research regarding these drivers.

## Review findings

**Fig. 1:**
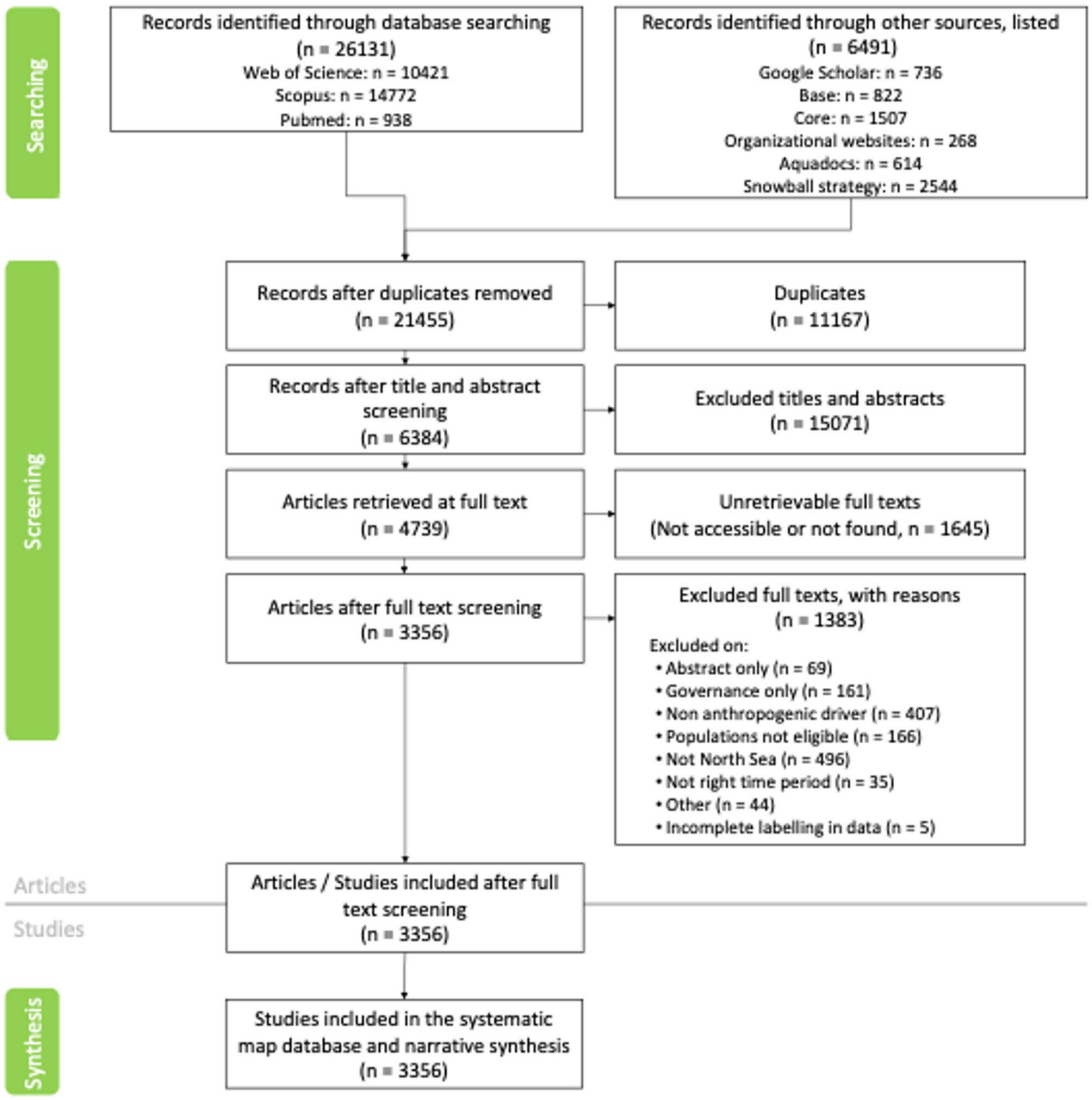
ROSES flow diagram showing the systematic mapping process [46]

The initial database search revealed a total of 31,016 articles from searches across WoS, Scopus, AquaDocs and Pubmed (in total n = 26,131), and through other sources (n = 6491) (Fig. 1).

Out of the total number of records retrieved, 10,111 duplicates and 15,071 articles were removed at the title and abstract screening stage, leaving 7440 articles. At the stage of retrieving full texts, 1645 articles were unretrievable (Additional file 4), leaving 5795 articles for the full-text screening process. An additional 2439 articles were excluded after the full-text screening due to duplication (n = 1056), non-fitting populations and comparators (n = 1379) (e.g., studies not within our North Sea domain) and not available (NAs = 4; no anthropogenic driver was selected during full text screening process). The latter compromised insufficient labeling by reviewers, leading to no information retrievable. Ultimately, a total of 3356 articles were included in the systematic map (Additional files 5, 6).

### Anthropogenic drivers over time and space

**Fig. 2:**
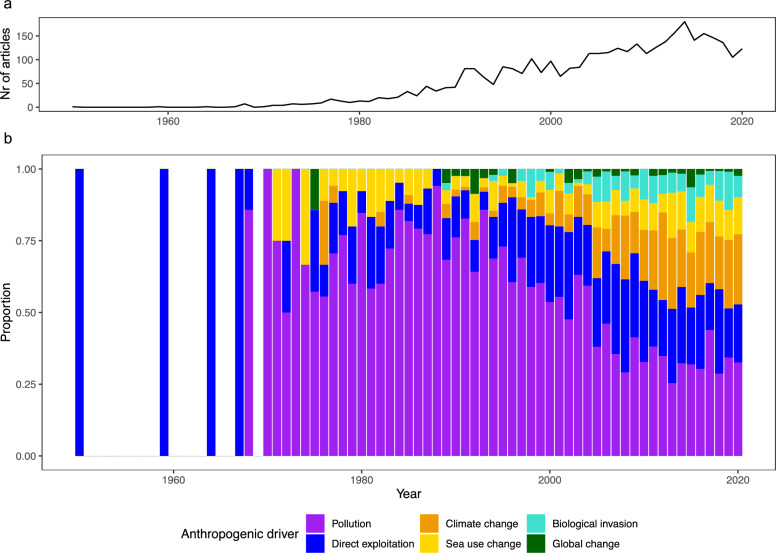
Number of articles per anthropogenic driver published over time (1945–2020). **a** total number of articles published per year over time; **b** proportion of anthropogenic driver published per year. Global change articles studied all five main drivers simultaneously

The analyzed time period of our study spans over 75 years (January 1945–December 2020). However, the first article meeting all criteria was found in 1950. The total number of published articles per year addressing the five main anthropogenic drivers increased strongly from the 1980s onwards (Fig. 2a). After a peak in 2014 (n = 180 articles), the number of publications per year decreased slightly until 2020 (n = 123). The first four articles in 1950, 1959, 1964 and 1967 were the only ones published in the respective years, and all of them were related to direct exploitation (Fig. 2b). From 1968 until the late 2000s, the focus of articles was mainly on the field of pollution, representing 85% (n = 6) and 94% (n = 32) in 1968 and 1988, respectively.

Articles on sea use change became more dominant in the 1970s, followed by a strong decrease in 1990 (0%). The number of articles in sea use change slightly increased again until 2020. Publications focusing on climate change increased each year from 1989 (4%, n = 2) until 2020 (24%, n = 16). Articles regarding biological invasion appeared consistently from 1997 (4%, n = 3) until 2020 (7%, n = 9), staying rather constant in numbers. Global change publications, that considered all five main drivers simultaneously, were mainly published last in the period, showing only a small number of publications from 1989 to 2020 (ranging between 0 and 8%). Over the 75 years analyzed, knowledge on pollution and direct exploitation was dominant throughout, whereas knowledge gaps were identified for drivers such as biological invasion and global change.

**Fig. 3:**
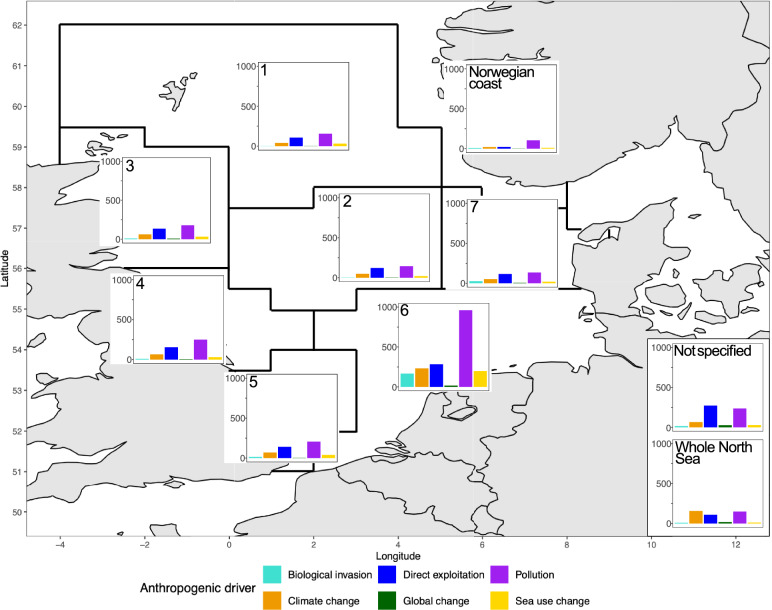
Number of anthropogenic drivers published in ICES (International Council for the Exploration of the Sea) standard species areas and the Norwegian coast. Global change articles studied all five main drivers simultaneously. Articles without any specific study area mentioned in the North Sea were categorized as ‘not specified’ while articles including study areas in the whole North Sea (areas IVa, b and c) were defined as ‘Whole North Sea’

The number of articles of anthropogenic drivers over space, using the ‘Whole North Sea’, the ‘ICES standard species areas (1–7)’, the ‘Norwegian coast’ and ‘not specified’ (studies where no specific location in the North Sea was mentioned) as categories, shows a similar pattern within the ICES areas (Fig. 3). Pollution represented the major proportion of articles in all ICES areas, followed by direct exploitation, climate change, sea use change, biological invasion, and lastly global change. It is apparent that most articles focused on ICES area 6 (n = 1858), whereas the least number of studies were carried out in the Norwegian coast area (n = 167). The distribution of articles per anthropogenic driver that focused on areas 1, 2, 3, and 7 (n = 338, n = 340, n = 422, n = 351, respectively) was similar but comparatively low to area 6. Areas 4 and 5 also showed similar patterns regarding the anthropogenic drivers, having a higher number of publications (n = 512 and n = 471, respectively) compared to 1, 2, 3 and 7. A different picture is seen for the categories ‘Whole North Sea’ and ‘not specified’. For the ‘Whole North Sea’, the strongest focus laid on climate change (34%, n = 157) and pollution (32%, n = 149). For ‘not specified’, the focus was highest for direct exploitation (42%, n = 272) and pollution (36%, n = 236). The majority of articles focusing on global change (4%, n = 642) were in the ‘not specified’ category and were not related to a specific area in the North Sea. Hence, there appear to be strong knowledge clusters regarding the southern North Sea regions, whereas less studies were conducted in northern regions, or considered the ‘Whole North Sea’.

### Distribution of anthropogenic drivers by drivers’ precision and study population

**Fig. 4:**
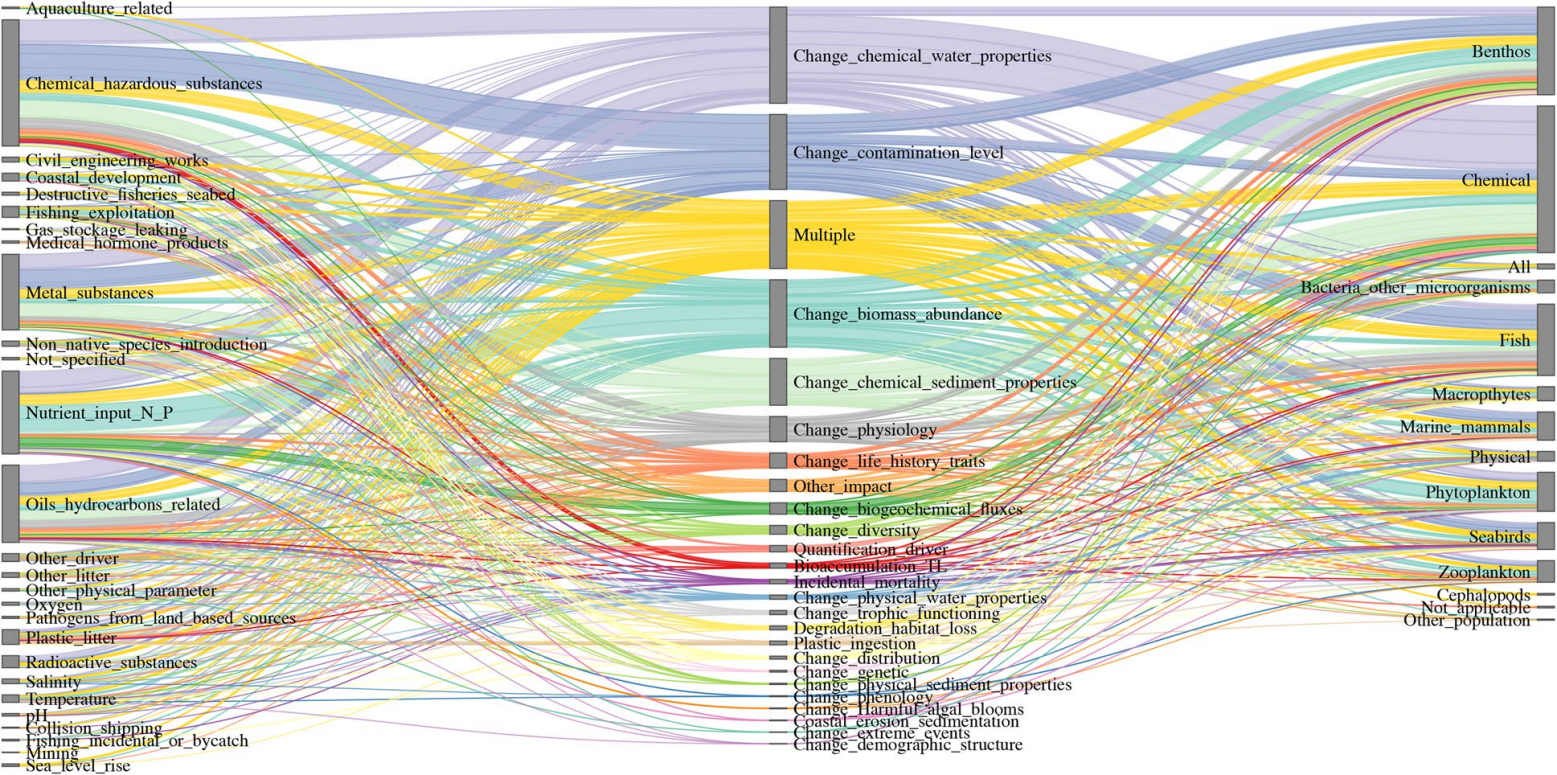
Sankey diagram for the anthropogenic driver pollution by analyzed impacts (middle row), divided into precision of driver (left row) and study population (right row). The nodes represent the respective entities, colored links show the flow between these, colors are accordingly to the entities in the middle row. The links’ width is proportional to the size of the flow to allow for comparison of contributions. Here, the reading direction is from the central entities towards the left and right, showing in how many driver precisions (left) the impacts (middle) are divided and which study population (right) the impacts affect

**Fig. 5:**
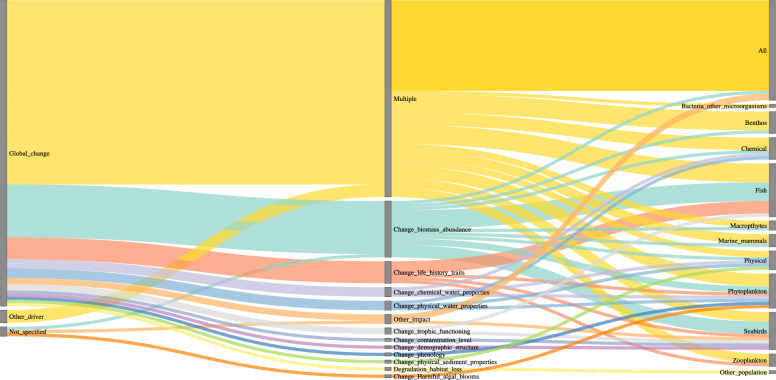
Sankey diagram for the anthropogenic driver global change by analyzed impacts (middle row), divided into precision of driver (left row) and study population (right row). Global change articles studied all five main drivers simultaneously. The nodes represent the respective entities, colored links show the flow between these, colors are accordingly to the entities in the middle row. The links’ width is proportional to the size of the flow to allow for comparison of contributions. Here, the reading direction is from the central entities towards the left and right, showing in how many driver precisions (left) the impacts (middle) are divided and which study population (right) the impacts affect

Articles showed differences in the ‘analyzed impacts’, the ‘precision of the drivers’ (more detail on the driver itself) and their relation to the ‘study population’, depending on the ‘anthropogenic driver’ investigated (Figs. 4, 5, Additional files 2). For biological invasion, climate change, direct exploitation and sea use change, the most analysed effect was change in biomass abundance. In contrast, for pollution, the dominant effects studied were changes in chemical water properties or changes in contamination level (Fig. 4). In third place, multiple impacts (more than three impacts) were analyzed with the pollution driver, whereas changes in biomass abundance were only fourth for this driver in the number of articles. The precision of the drivers regarding pollution were strongly focusing on chemical substances, such as hazardous substances, metal substances, eutrophication (nutrient input of nitrogen and phosphorous in the environment), as well as oil and hydrocarbon related substances. The effects of these precise drivers and changes in chemical water properties were mainly studied on the chemical environment, benthos and fish.

Regarding pollution, knowledge clusters exist for studies that focus on changes in the chemical water properties (e.g., chemicals in dissolved water) and to a lesser extent on changes in contamination level in benthos and fish. Even fewer studies included analyses on other study populations like seabirds, zooplankton or, to an even lesser extent cephalopods or bacteria, highlighting clear knowledge gaps for these trophic groups.

Literature focusing on global change included all five drivers and had a strong tendency towards analyzing multiple impacts (more than three impacts) and their effects on all study populations (Fig. 5). These articles included to a high share reports, which provide a broad overview of drivers affecting ecosystems and their populations. Even though individual species were mentioned, our screening method allows for the category ‘all’ only if all study populations are represented. This way, distinct study populations were also, to a varying degree, included in the ‘all’ label. Notably, articles focusing on global change, along with climate change (temperature), were the only ones with a strong focus on phytoplankton and seabirds. Fish were mainly studied in articles related to direct exploitation. Sea use change, on the other hand, had the highest variability in study populations investigated. The major precise drivers were civil engineering works and coastal development (e.g., construction of offshore wind farms), which contributed most to changes in biomass. Knowledge clusters are still visible for the abiotic (chemical, physical) levels, fish, higher trophic levels (seabirds, marine mammals), and benthos. The strongest analyzed impacts on benthos were through changes in biomass and abundance that occurred through the introduction of non-native species. Also, macrophytes were most frequently studied within the context of biological invasion, compared to the other anthropogenic drivers. The analyses of the anthropogenic drivers, analyzed impacts, the precision of these drivers and the study population investigated revealed clear knowledge clusters and knowledge gaps. In general, a lot of knowledge is provided for changes in impacts on biomass and abundance and on multiple impacts on benthos and fish, highlighting knowledge clusters. Still, the analyses revealed knowledge gaps for macrophytes, zooplankton, especially cephalopods and bacteria and other microorganisms.

### Distribution of anthropogenic drivers by organizational level

**Fig. 6:**
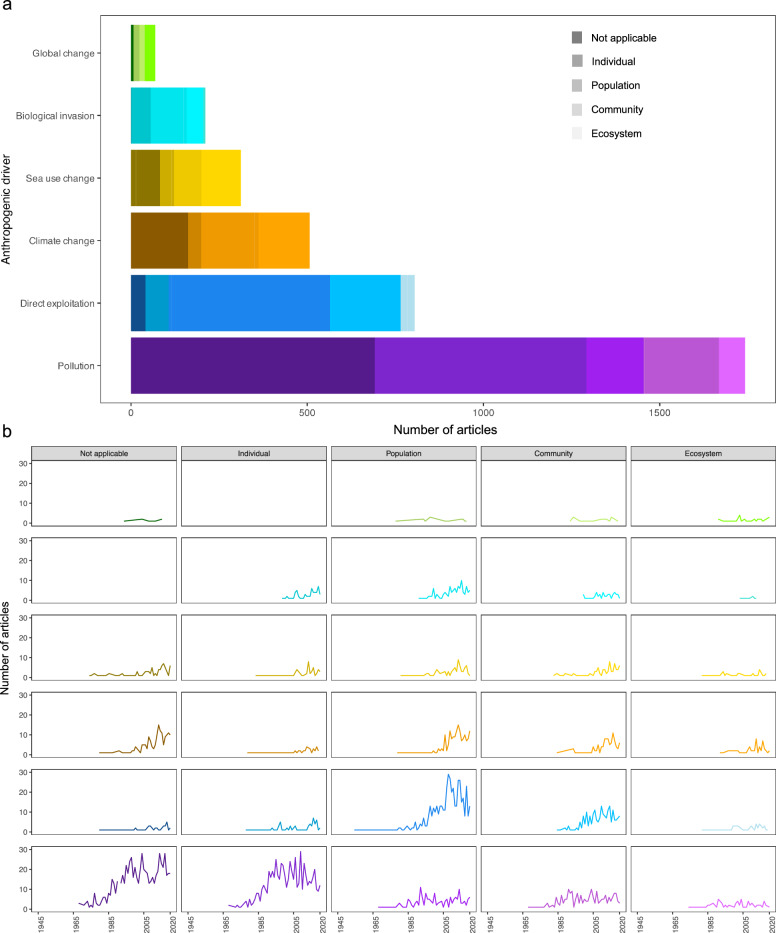
Total number of articles per organizational level per anthropogenic driver over time (1945–2020). **a** Published articles per organizational level; **b** number of articles published per organizational level per year. Global change articles studied all five main drivers simultaneously

We distinguished four organizational levels: individual (only a single individual considered), population (e.g., fish stock), community (e.g., fish community) and ecosystem level (e.g., Wadden Sea and all its compartments, meaning living organisms and abiotic factors). The distribution of organizational levels among the anthropogenic drivers varied greatly (Fig. 6a). Most studies lacking a specified organizational level were related to pollution (39%, n = 692/1742) mostly focusing on chemical and metal substances, whereas there was only one article with no organizational level for biological invasion (0.04%, n = 1/211). On an individual level, most articles were also published within pollution (34%, n = 600/1742), and the least within climate change topics (7%, n = 37/507). However, on a population level, pollution had the lowest share (9%, n = 162/1742) and direct exploitation the highest (56%, n = 457/805). Communities were mostly studied in the context of sea use change (25%, n = 80/312) and the least in the context of pollution (12%, n = 214/1742). Global change, where all five drivers are considered at once, had the highest proportion in ecosystems (43%, n = 30/69), whereas biological invasion had the lowest (2%, n = 6/211). Physical and chemical pollutions were mainly studied through the assessment of the abiotic components (chemical and physical water properties) of the North Sea ecosystem (Fig. 4). Hence, knowledge regarding pollution was not as available at population and community levels. These were rather explored for sea use change and global change, respectively. Global change included many reports that highlight all anthropogenic drivers and their effects on multiple dimensions and study populations (Fig. 5). This way, ecosystems, demonstrating a strong interaction between the abiotic (physical, chemical) and biotic compartments, included the full holistic dynamics driven by global change. Populations mainly studied in direct exploitation articles relate to fishing exploitation on fish populations.

The amount of articles differed strongly between anthropogenic drivers and organizational level over the study period (Fig. 6b). While the number of articles on global change remained almost constant, there were slight increases in the late 2000s for biological invasion, sea use change and climate change. Direct exploitation articles increased strongly at a ‘population’ level in the 1990s, and to a lesser extent at a community level since the 2000s. The ‘not applicable’ and ‘individual’ levels had the highest records in pollution articles, both raised intensively since the 1980s. Overall, there is a knowledge gap regarding global change at all organizational levels. Studies on ecosystems in general were comparatively low in contrast to individual and population levels for pollution and direct exploitation (knowledge clusters).

### Distribution of methodologies among anthropogenic drivers

**Fig. 7:**
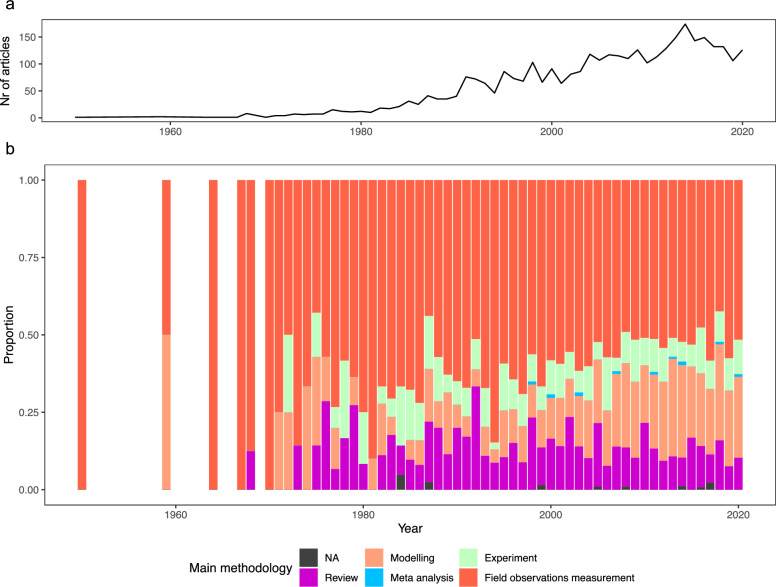
Number of main methodologies used in the published articles over time (1945–2020). **a** total number of articles published per year over time; **b** proportion of methodology used published per year. Global change articles studied all five main drivers simultaneously

The methodologies used in the articles change in proportion over time (Fig. 7). Sometimes more than one methodology was used in the same article, e.g., the article in 1959 includes field observations measurement and modelling (Fig. 7b). Across the whole time period, the biggest proportion was made up of field observations measurement, being continuously implemented each year in our study period (1950: 100%, n = 1, and 2020: 51%, n = 65). However, the share of field observations measurement decreased over time, whereas modelling increased. Articles involving experiments and reviews had lower proportions and only appeared consistently each year from 1982 onwards, at which point the overall number of papers published per year increased steadily (Fig. 2). Meta-analyses occurred the least (0 – 1%, n = 2 in 2014).

**Fig. 8:**
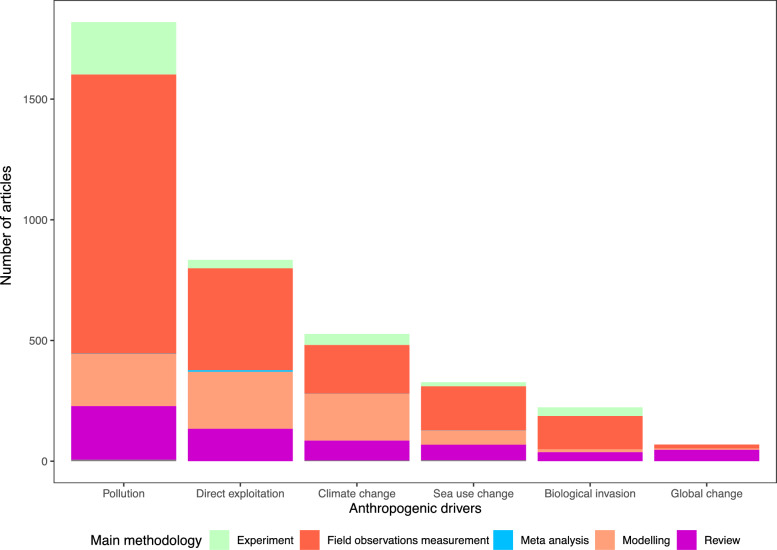
Number of articles per anthropogenic driver and main methodology. Global change articles studied all five main drivers simultaneously

Differences in the methodologies applied in the articles were evident across anthropogenic drivers (Fig. 8). All five methodologies were used only in articles focusing on pollution, direct exploitation, and sea use change. No meta-analyses studies related to climate change, biological invasion and global change were found, with the latter also lacking experimental studies. On the other hand, global change articles had the highest overall proportion of reviews (68%, n = 47/69). Field observations measurements was the method applied most for the other anthropogenic drivers, followed by modelling, reviews and experiments. Only climate change articles included field observations measurements (38%, n = 201/572) and modelling (36%, n = 194/572) to an almost equal share.

In contrast, meta-analyses were by far the least represented (up to 0.08% maximum). In total, 13 NAs were included in the analyses, were no labeling occurred for the method during the full-text screening process. In general, it is obvious that knowledge clusters exist around articles applying field observations measurements. The proportion of field observations measurement, modeling and review was almost evenly distributed among the anthropogenic drivers, showing that meta-analyses and experiments were not the preferred methodologies. With the increasing threat of climate change to ecosystems in mind, it would be expected that modelling represents a comparatively high share in the future due to forecasting models.

### Limitations of the map

While the search strategy used to create this systematic map was designed to encompass a wide range of anthropogenic drivers in the North Sea, it is not comprehensive, and we recognize potential for limitations. We have included a wide range of areas in our search string, but probably missed locally performed studies at certain locations. Also, the search terms focus strongly on the five main anthropogenic drivers, leaving space for undetected impacts that are not covered in detail, such as e.g., tourism and landscape changes causing loss of coastal protection. Furthermore, based on the broad taxonomic resolution this study is not able to highlight important threats for certain species that play a key role in the North Sea. For example, jellyfish were categorized as zooplankton, just like copepods. With these limitations, we may have missed important threats for certain species that play a key role in North Sea areas. Nevertheless, we are confident that our search terms cover all threats that emerges out of the five main anthropogenic drivers.

Although our map provides evidence on the whole North Sea, several articles were excluded due to language barriers, e.g., Dutch, Italian, Spanish publications. In this way, especially nationally published reports on North Sea research were missed. Also, we see a steep increase of articles over time, especially since the 1980s. The low number of papers at the beginning of the time series could be a bias due to lacking digitalization. Articles found in the earlier decades (1950s to ~ 1980s) were often a scanned version of formerly printed articles. Hence, the results of our study are strongly dependent on the number of articles being available electronically over time. Although the participating reviewers screened the full texts throughout, the final data set contained 17 NAs where no information was added to certain labels during the screening. Still, given the large number of texts screened, we do not expect any major effects on the results of our study. Furthermore, we did not update our search to the most recent years due to the lack of time and manpower. However, given our 75-year span (January 1945–December 2020), we believe that the exclusion of the last four years would not strongly affect our results, particularly as this represents the most extensive systematic map to date. This is especially relevant considering the global slowdown in research activities during that period due to the COVID-19 pandemic, which limited research activities worldwide (e.g., the conduction of experiments and field observations) [49, 51, 56, 57]. Given the detailed description of our map in our protocol [42] and in the methods section of the current paper, the search for more recent years can be updated in the future. These updates can thus benefit this systematic map.

## Conclusions

This systematic map provides the first comprehensive overview of research trends over 75 years (January 1945–December 2020) concerning the top five anthropogenic drivers affecting marine ecosystem changes in the North Sea: direct exploitation of fish and seafood, sea use change, human-driven climate change, pollution, and invasive alien species. We found that there were a high number of studies in the field of pollution and an increasing interest in direct exploitation and climate change topics, and in their effects, on fish and benthos biomass especially. However, sea use changes and biological invasion drew less research interest during the study period. We also identified knowledge gaps for global change studies that considered all five main anthropogenic drivers at once and in spatial coverage in the studies, with a higher proportion of studies being performed in the southern North Sea areas. Studies were mainly performed through field observations measurements, whereas meta-analyses for a holistic approach are scarce.

### Implications for policy/management

Effective management of marine anthropogenic drivers is becoming more important than ever due to increasing threats to ecosystem resilience and biodiversity [4–8, 8–59, 59–68]. The need to understand, assess and mitigate anthropogenic drivers is increasing in EU member states, especially in the context of global goals like the SDG goals of the United Nations (SDG 14—Life below water) [31], and the UN Decade of Ocean Science [35–38], as well as within the EU level context implementing ICES advices and OSPAR [28, 40, 41]. Given the changes in the strength of the drivers studied in the North Sea over time, EU management is required to adapt quickly to differences in drivers’ importance. While direct exploitation through fishing, for instance, has historically led to substantial declines in the North Sea fish populations over five decades [7, 8], the effects of climate change prevent population recovery despite current fishing pressure reductions [54, 58, 60, 61]. Knowing that climate change is expected to outpace the other drivers [13–15], a flexible and adaptable management approach is needed. Rapid changes in populations, so-called regime shifts, are well known in the North Sea for lower trophic levels, caused by climate change [56, 57, 59, 62, 64], and in fish populations, as a result of the interplay between direct exploitation and climate change induced water temperature increases [21, 54, 58, 60, 61, 65]. Climate change also causes changes in species distribution due to changes in thermal habitats [28, 62, 65, 66, 69], which could not be determined with our systematic map showing knowledge clusters in the southern North Sea regions while the northern North Sea is less considered. Changes in species distribution make it difficult to transfer knowledge between different areas due to changes in ecosystem structures [21, 70]. Decision-makers should therefore be aware of this and take caution when transferring and generalizing results from one spatial area to another [66, 71].

An ecosystem-based management to reach sustainable (development) goals requires us to understand linkages between different systems. Our map allows decision-makers to understand system changes within the field of a certain anthropogenic driver. However, our map also revealed the lack of information on global changes, including the impacts of all drivers at the same time, and their coherent effects at an ecosystem level thus diminishing the opportunity for decision-makers to include cascading effects and possible repercussions that pass through several systems (e.g., study population level: trophic levels; ecosystem level: different habitats vertically and horizontally). Such lack of evidence hinders a holistic ecosystem management approach, as required by OSPAR [23, 53, 67]. Furthermore, offshore wind farms (OWF), among other energy infrastructure, are currently implying the most structural change due to the EU’s Green Deal. Our map has identified a knowledge gap regarding the novelty of OWF development and their impacts on the environment [24, 25]. Hence, the change in importance of the drivers for the North Sea ecosystems in the future should be taken into account by decision-makers for a sustainable management. Decision-makers should commission further systematic maps and reviews in the rapidly evolving field of climate change to better determine its impacts on study populations. It would prevent missing out rapid developments and allow to assess cascading effects and repercussions for humans and their well-being [68, 69, 72, 73]. Especially given the currently on-going 7th assessment cycle (AR7) by IPCC, further climate change and marine research is demanded to inform decision-makers and provide decision-support tools about climate change variables, ocean processes, and potential adaptation strategies, as outlined by their working groups [70, 74].

Our systematic map represents a useful resource to understand (i) the effects of the five main anthropogenic drivers on the North Sea system (complementing to the knowledge gaps identified by [71, 75]; taking chemical and biological effects of man-made structures into account), (ii) knowledge clusters which can be helpful for decision-making to implement appropriate measures, (iii) knowledge gaps for potential further research, and (iv) potential developments of methodologies applied to study the drivers.

### Implications for research

This systematic map has uncovered a disproportional research focus in understanding the top five anthropogenic drivers (direct exploitation of fish and seafood, sea use change, human-driven climate change, pollution, and invasive alien species) and their environmental effects in the North Sea from 1945 to 2020. High research investment has been put into understanding pollution, even though climate change appears to be the driver that will become most important in future [13–15]. Often, chemical water properties were studied in the southern North Sea, neglecting detailed information regarding pollution of oil platforms located in the northern North Sea. In general, evidence on the drivers is unevenly distributed across the North Sea. Clear knowledge gaps exist in the northern parts, where more research is required to gain a comprehensive assessment of drivers’ effects on ecosystems. Additionally, the species studied were clearly disproportionate, with macrophytes, zooplankton, cephalopods, and bacteria and other microorganisms being studied the least. This might have strong implications, especially because microorganisms are essential for underlying biogeochemical processes. They provide high catalytic capacities, building the basis for higher trophic life [72]. Hence, more research at other population levels is needed for understanding linkages between trophic levels, either positive or negative and causing system changes if they diminish and if new ones establish their populations. Studies on specific drivers were mainly performed through field measurements and modelling methodologies. Often, studies focus only on single drivers without inter-relations, cumulative effects or feedback mechanisms of drivers. Studies linking the results of already published work, e.g., using reviews and meta-analyses are comparatively low, but are needed to understand system linkages.

The distribution in knowledge clusters revealed in this strong evidence based systematic map align with the results of areas worldwide. Given the increasing impact of climate change, the field is rapidly evolving, filling knowledge gaps worldwide [68, 69, 72, 73, 76]. Also the number in published articles in biological invasions generally expanded over the past decades, showing a strong research interest in this field. However, in the case of adjacent North Sea areas, the strength of studied impacts might slightly differ at regional scales—as for local differences within the North Sea. In the Norwegian region, aquaculture around salmon is steadily increasing, being the worldwide second biggest aquaculture industry nowadays. Hence, increased research on direct exploitation of fish and seafood (aquaculture) can be expected here [74–79]. In the Baltic Sea, research largely directs towards fishing, climate change and pollution (eutrophication). Here, these drivers are interlinked through the strong exploitation of fish stocks that determine coastal inhabitants’ livelihood. Key species were overfished until depletion and are not recovering due to rising climate change and eutrophication effects [79–85]. Even though regional and local areas might have different outcomes in the strength of emergent of fields, climate change appears still to be the major driver being studied in the future [68, 72]. Continuously incorporating recent studies in this map can further prove these findings, as well as address further affected ecosystems compartments that were not determined yet.

In general, future research and systematic maps can evolve on this basis. On the one hand, identified knowledge gaps provide key opportunities for future research to take studies at different spatial scales into account. Since this systematic map highlights ecological effects of anthropogenic drivers, future research could include a multidisciplinary team to analyze interlinkages between ecological, social and economic dimensions to further support the Sustainable Development Goals. The evidence base also provides possibilities to draw linkages between anthropogenic drivers and their effects on study population, as many studies included several impacts simultaneously. Researchers can also deepen knowledge clusters, such as analyzing drivers’ impacts on species and at local levels of the North Sea and adjacent seas for comparison reasons. Future research can focus on which areas are impacted most and whether management implications are necessary for restoration.

In a world facing escalating threats from anthropogenic drivers on ecosystems, understanding interactions between the human activities and natural systems is more crucial than ever [84, 85]. To meet the goals of sustainable use of marine ecosystems, it is important to study feedback mechanisms between drivers, as well as their synergistic and antagonistic effects. Only a holistic perspective, with articles assessing global changes, allows us to understand, adapt, and mitigate anthropogenic drivers in the future.

## Supplementary Information

Additional File 1: ROSES form, description: filled in ROSES form

Additional File 2: Complete Search Results

Additional File 3: Label description of the map, description: the file shows explicit descriptionsof all labels that were used during the coding phase.

Additional File 4: Unobtainable full texts, description: the file contains a list with all full textsthat were unretrievable

Additional File 5: List of articles included and answers of screening process, description: thefile provides an overview of all answers that were provided during the screening process. Thisfile was used for the final analyses.

Additional File 6: List of articles excluded after full text screening, description: the fileprovides an overview of all articles that were excluded during the screening process and showstheir exclusion criteria.

## Data Availability

The coding guide, datasets generated and analyzed during the current study, as well as the R codes for analyses and figures are freely available in the “north_sea_review” repository, https://github.com/HeleneGutte/north_sea_review.

## References

[1] Díaz S, Settele J, Brondízio ES, Ngo HT, Agard J, Arneth A, et al. Pervasive human-driven decline of life on Earth points to the need for transformative change. Science. 2019;366:eaax3100. 31831642 10.1126/science.aax3100

[2] Butchart SHM, Walpole M, Collen B, Van Strien A, Scharlemann JPW, Almond REA, et al. Global biodiversity: indicators of recent declines. Science. 1979;2010(328):1164–8. 10.1126/science.1187512 20430971

[3] Di Minin E, Brooks TM, Toivonen T, Butchart SHM, Heikinheimo V, Watson JEM, et al. Identifying global centers of unsustainable commercial harvesting of species. Sci Adv. 2019;5:eaau2879. 30949571 10.1126/sciadv.aau2879 PMC6447386

[4] Cardinale BJ, Duffy JE, Gonzalez A, Hooper DU, Perrings C, Venail P, et al. Biodiversity loss and its impact on humanity. Nature. 2012;486:59–67. 22678280 10.1038/nature11148

[5] Steffen W, Broadgate W, Deutsch L, Gaffney O, Ludwig C. The trajectory of the anthropocene: the great acceleration. Anthropocene Rev. 2015;2:81–98.

[6] Hammerschlag N, Schmitz OJ, Flecker AS, Lafferty KD, Sih A, Atwood TB, et al. Ecosystem function and services of aquatic predators in the anthropocene. Trends Ecol Evol. 2019;34:369–83. 30857757 10.1016/j.tree.2019.01.005

[7] Jouffray JB, Blasiak R, Norström AV, Österblom H, Nyström M. The blue acceleration: the trajectory of human expansion into the ocean. One Earth. 2020;2:43–54.

[8] Gissi E, Manea E, Mazaris AD, Fraschetti S, Almpanidou V, Bevilacqua S, et al. A review of the combined effects of climate change and other local human stressors on the marine environment. Sci Total Environ. 2021. 10.1016/j.scitotenv.2020.142564. 10.1016/j.scitotenv.2020.142564 33035971

[9] Halpern BS, Longo C, Stewart Lowndes JS, Best BD, Frazier M, Katona SK, et al. Patterns and emerging trends in global ocean health. PLoS ONE. 2015;10:e0117863. 25774678 10.1371/journal.pone.0117863 PMC4361765

[10] Halpern BS, Frazier M, Potapenko J, Casey KS, Koenig K, Longo C, et al. Spatial and temporal changes in cumulative human impacts on the world’s ocean. Nat Commun. 2015;6:1–7. 10.1038/ncomms8615 PMC4510691 26172980

[11] Stock A, Crowder LB, Halpern BS, Micheli F. Uncertainty analysis and robust areas of high and low modeled human impact on the global oceans. Conserv Biol. 2018;32:1368–79. 29797608 10.1111/cobi.13141

[12] Reid WV, Mooney HA, Cropper A, Capistrano D, Carpenter SR, Chopra K, et al. Ecosystems and human well-being—synthesis: a report of the Millennium Ecosystem Assessment. Washington: Island Press; 2005.

[13] IPBES. Global assessment report on biodiversity and ecosystem services of the Intergovernmental Science-Policy Platform on Biodiversity and Ecosystem Services. Bonn; 2019.

[14] Ceballos G, Ehrlich PR, Barnosky AD, García A, Pringle RM, Palmer TM. Accelerated modern human-induced species losses: entering the sixth mass extinction. Sci Adv. 2015;1:e1400253. 26601195 10.1126/sciadv.1400253 PMC4640606

[15] Arneth A, Shin Y-J, Leadley P, Rondinini C, Bukvareva E, Kolb M, et al. Post-2020 biodiversity targets need to embrace climate change. Proc Natl Acad Sci. 2020;117:30882–91. 33288709 10.1073/pnas.2009584117 PMC7739876

[16] Bellard C, Bertelsmeier C, Leadley P, Thuiller W, Courchamp F. Impacts of climate change on the future of biodiversity. Ecol Lett. 2012;15:365–77. 22257223 10.1111/j.1461-0248.2011.01736.x PMC3880584

[17] Gattuso JP, Magnan A, Billé R, Cheung WWL, Howes EL, Joos F, et al. Contrasting futures for ocean and society from different anthropogenic CO2 emissions scenarios. Science (1979). 2015;349:aac4722. 10.1126/science.aac4722 26138982

[18] Lotze HK, Tittensor DP, Bryndum-Buchholz A, Eddy TD, Cheung WWL, Galbraith ED, et al. Global ensemble projections reveal trophic amplification of ocean biomass declines with climate change. Proc Natl Acad Sci USA. 2019;116:12907–12. 31186360 10.1073/pnas.1900194116 PMC6600926

[19] Cheung WWL, Lam VWY, Sarmiento JL, Kearney K, Watson R, Pauly D. Projecting global marine biodiversity impacts under climate change scenarios. Fish Fish. 2009;10:235–51.

[20] Kuparinen A, Boit A, Valdovinos FS, Lassaux H, Martinez ND. Fishing-induced life-history changes degrade and destabilize harvested ecosystems. Sci Rep. 2016;6:1–9. 26915461 10.1038/srep22245 PMC4768105

[21] Halpern BS, Walbridge S, Selkoe KA, Kappel CV, Micheli F, D’Agrosa C, et al. A global map of human impact on marine ecosystems. Science. 1979;2008(319):948–52. 10.1126/science.1149345 18276889

[22] IPBES. The IPBES regional assessment report on biodiversity and ecosystem services for Europe and Central Asia. Bonn; 2018.

[23] OSPAR. Quality status report. London; 2010.

[24] Daewel U, Akhtar N, Christiansen N, Schrum C. Offshore wind farms are projected to impact primary production and bottom water deoxygenation in the North Sea. Commun Earth Environ. 2022;3:292.

[25] Gușatu LF, Menegon S, Depellegrin D, Zuidema C, Faaij A, Yamu C. Spatial and temporal analysis of cumulative environmental effects of offshore wind farms in the North Sea basin. Sci Rep. 2021;11:10125. 33980905 10.1038/s41598-021-89537-1 PMC8115305

[26] Pecl GT, Araújo MB, Bell JD, Blanchard J, Bonebrake TC, Chen I-C, et al. Biodiversity redistribution under climate change: impacts on ecosystems and human well-being. Science. 2017;355:eaai9214. 28360268 10.1126/science.aai9214

[27] Urban MC. Accelerating extinction risk from climate change. Science. 1979;2015(348):571–3. 10.1126/science.aaa4984 25931559

[28] Emeis KC, Van Beusekom J, Callies U, Ebinghaus R, Kannen A, Kraus G, et al. The North Sea—a shelf sea in the Anthropocene. J Mar Syst. 2015;141:18–33.

[29] Burrows MT, Schoeman DS, Buckley LB, Moore P, Poloczanska ES, Brander KM, et al. The pace of shifting climate in marine and terrestrial ecosystems. Science. 1979;2011(334):652–5. 10.1126/science.1210288 22053045

[30] Ramírez F, Afán I, Davis LS, Chiaradia A. Climate impacts on global hot spots of marine biodiversity. Sci Adv. 2017;3:e1601198. 28261659 10.1126/sciadv.1601198 PMC5321448

[31] Hoegh-Guldberg O, Northrop E, Lubchenco J. The ocean is key to achieving climate and societal goals. Science. 1979;2019(365):1372–4. 10.1126/science.aaz4390 31554733

[32] Duarte CM. Global change and the future ocean: a grand challenge for marine sciences. Front Mar Sci. 2014;1:63.

[33] Heymans JJ, Bundy A, Christensen V, Coll M, de Mutsert K, Fulton EA, et al. The Ocean Decade: a true ecosystem modeling challenge. Front Mar Sci. 2020;7:554573.

[34] IPBES. The methodological assessment report on scenarios and models of biodiversity and ecosystem services. Bonn, Germany; 2016.

[35] Ryabinin V, Barbière J, Haugan P, Kullenberg G, Smith N, McLean C, et al. The UN decade of ocean science for sustainable development. Front Mar Sci. 2019. 10.3389/fmars.2019.00470.

[36] Wisz MS, Satterthwaite EV, Fudge M, Fischer M, Polejack A, St. John M, et al. 100 opportunities for more inclusive ocean research: cross-disciplinary research questions for sustainable ocean governance and management. Front Mar Sci. 2020;7:541934.

[37] Claudet J, Bopp L, Cheung WWL, Devillers R, Escobar-Briones E, Haugan P, et al. A roadmap for using the UN decade of ocean science for sustainable development in support of science, policy, and action. One Earth. 2020;2:34–42.

[38] Degraer S, Van Lancker V, Van Dijk TAGP, Birchenough SNR, De Witte B, Elliott M, et al. Interdisciplinary science to support North Sea marine management: lessons learned and future demands. Hydrobiologia. 2019;845:1–11.

[39] Mazor T, Doropoulos C, Schwarzmueller F, Gladish DW, Kumaran N, Merkel K, et al. Global mismatch of policy and research on drivers of biodiversity loss. Nat Ecol Evol. 2018;2:1071–4. 29784980 10.1038/s41559-018-0563-x

[40] Stenseth NC, Payne MR, Bonsdorff E, Dankel DJ, Durant JM, Anderson LG, et al. Attuning to a changing ocean. Proc Natl Acad Sci USA. 2020;117:20363–71. 32817527 10.1073/pnas.1915352117 PMC7456143

[41] ICES. Greater North Sea ecosystem overview. 2024.

[42] Moullec F, Asselot R, Auch D, Blöcker AM, Börner G, Färber L, et al. Identifying and addressing the anthropogenic drivers of global change in the North Sea: a systematic map protocol. Environ Evid. 2021;10:1–11. 10.1186/s13750-025-00377-2 PMC12673759 41331668

[43] ICES. SISP 10—Manual for the North Sea international bottom trawl surveys. Copenhagen; 2020.

[44] Trifonova N, Kenny A, Maxwell D, Duplisea D, Fernandes J, Tucker A. Spatio-temporal Bayesian network models with latent variables for revealing trophic dynamics and functional networks in fisheries ecology. Ecol Inform. 2015;30:142–58.

[45] CEE. Guidelines and standards for evidence synthesis in environmental management. Version 5.1 [Internet]. 2022 [cited 2024 May 3]. https://environmentalevidence.org/information-for-authors/

[46] Haddaway NR, Macura B, Whaley P, Pullin AS. ROSES reporting standards for systematic evidence syntheses: pro forma, flow-diagram and descriptive summary of the plan and conduct of environmental systematic reviews and systematic maps. Environ Evid. 2018;7:1–8.

[47] Bozada T, Borden J, Workman J, Del Cid M, Malinowski J, Luechtefeld T. Sysrev: a FAIR platform for data curation and systematic evidence review. Front Artif Intell. 2021;4:685298. 34423285 10.3389/frai.2021.685298 PMC8374944

[48] Wickham H, Averick M, Bryan J, Chang W, D’Agostino Mcgowan L, François R, et al. Welcome to the Tidyverse. J Open Source Softw. 2019;4:1686.

[49] Madhusoodanan J. Pandemic productivity dip may linger. Science. 1979;2021:519–519. 10.1126/science.acx9467 34709888

[50] R Core Team. R: A language and environment for statistical computing. R Foundation for Statistical Computing. 2018.

[51] Raynaud M, Goutaudier V, Louis K, Al-Awadhi S, Dubourg Q, Truchot A, et al. Impact of the COVID-19 pandemic on publication dynamics and non-COVID-19 research production. BMC Med Res Methodol. 2021;21:1–10. 10.1186/s12874-021-01404-9. 34809561 10.1186/s12874-021-01404-9 PMC8607966

[52] Wickham H. Ggplot2: elegant graphics for data analysis. New York: Springer-Verlag; 2016.

[53] Allaire JJ, Ellis P, Gandrud C, Kuo K, Lewis BW, Owen J, et al. Package “networkD3.” D3 JavaScript network graphs from R. 2017

[54] Blöcker AM, Sguotti C, Möllmann C. Discontinuous dynamics in North Sea cod Gadus morhua caused by ecosystem change. Mar Ecol Prog Ser. 2023;713:133–49.

[55] Gerritsen H. Package “mapplots.” 2018

[56] Fromentin J-M, Planque B. *Calanus* and environment in the eastern North Atlantic. II. Influence of the North Atlantic Oscillation on *C. finmarchicus* and *C. helgolandicus*. Mar Ecol Prog Ser. 1996;134:111–8.

[57] Alheit J, Möllmann C, Dutz J, Kornilovs G, Loewe P, Mohrholz V, et al. Synchronous ecological regime shifts in the central Baltic and the North Sea in the late 1980s. ICES J Mar Sci. 2005;62:1205–15.

[58] Blöcker AM, Gutte HM, Bender RL, Otto SA, Sguotti C, Möllmann C. Regime shift dynamics, tipping points and the success of fisheries management. Sci Rep. 2023;13:289. 36609587 10.1038/s41598-022-27104-y PMC9822959

[59] Reid PC, Hari RE, Beaugrand G, Livingstone DM, Marty C, Straile D, et al. Global impacts of the 1980s regime shift. Glob Chang Biol. 2016;22:682–703. 26598217 10.1111/gcb.13106 PMC4738433

[60] Sguotti C, Otto SA, Cormon X, Werner KM, Deyle E, Sugihara G, et al. Non-linearity in stock–recruitment relationships of Atlantic cod: insights from a multi-model approach. ICES J Mar Sci. 2020;77:1492–502.

[61] Sguotti C, Blöcker A, Färber L, Blanz B, Cormier R, Diekmann R, et al. Irreversibility of regime shifts in the North Sea. Front Mar Sci. 2022;1830:945204.

[62] Engelhard GH, Pinnegar JK, Kell LT, Rijnsdorp AD. Nine decades of North Sea sole and plaice distribution. ICES J Mar Sci. 2011;68:1090–104.

[63] Engelhard GH, Righton DA, Pinnegar JK. Climate change and fishing: a century of shifting distribution in North Sea cod. Glob Chang Biol. 2014;20:2473–83. 24375860 10.1111/gcb.12513 PMC4282283

[64] Baudron AR, Brunel T, Blanchet M-A, Hidalgo M, Chust G, Brown EJ, et al. Changing fish distributions challenge the effective management of European fisheries. Ecography. 2020;43:494–505.

[65] Lenoir J, Bertrand R, Comte L, Bourgeaud L, Hattab T, Murienne J, et al. Species better track climate warming in the oceans than on land. Nat Ecol Evol. 2020;4:1044–59. 32451428 10.1038/s41559-020-1198-2

[66] Paxton AB, Foxfoot IR, Cutshaw C, Steward DN, Poussard L, Riley TN, et al. Evidence on the ecological and physical effects of built structures in shallow, tropical coral reefs: a systematic map. Environ Evid. 2024;13:12. 39294693 10.1186/s13750-024-00336-3 PMC11378790

[67] OSPAR. Homepage: convention for the protection of the marine environment of the North-East Atlantic [Internet]. 2024 [cited 2024 May 15]. https://www.ospar.org/

[68] Li C, Yao H, Li Z, Wu F, Liu B, Wu Y, et al. A bibliometric analysis of global research on climate change and agriculture from 1985 to 2023. Agronomy. 2024;14:2729. 10.3390/agronomy14112729.

[69] Tautiva JAD, Huaman J, Ponce Oliva RD. Trends in research on climate change and organizations: a bibliometric analysis (1999–2021). Management Review Quarterly [Internet]. 2022 [cited 2025 Apr 10];74:227–61. Available from: https://link.springer.com/article/10.1007/s11301-022-00298-1

[70] IPCC. Seventh Assessment Report [Internet]. 2025 [cited 2025 Apr 10]. Available from: https://www.ipcc.ch/assessment-report/ar7/

[71] Lemasson AJ, Somerfield PJ, Schratzberger M, Mcneill CL, Nunes J, Pascoe C, et al. Evidence for the effects of decommissioning man-made structures on marine ecosystems globally: a systematic map. Environ Evid. 2022;11:35. 39294784 10.1186/s13750-022-00285-9 PMC11378805

[72] Jorgensen BB. Bacteria and marine biogeochemistry. Marine Geochemistry [Internet]. Springer Berlin Heidelberg; 2000 [cited 2024 Jun 12]. p. 173–207. https://www.researchgate.net/publication/226160230_Bacteria_and_Marine_Biogeochemistry

[73] Fu HZ, Waltman L. A large-scale bibliometric analysis of global climate change research between 2001 and 2018. Clim Change. 2022;170:36. 10.1007/s10584-022-03324-z.

[74] Naylor R, Fang S, Fanzo J. A global view of aquaculture policy. Food Policy. 2023;116:102422.

[75] FAO. Fishery and Aquaculture Statistics. Global aquaculture production 1950–2020 (FishStatJ) [Internet]. FAO Fisheries and Aquaculture Division. 2022 [cited 2025 Apr 10]. https://www.fao.org/fishery/en/statistics/software/fishstatj

[76] Iversen A, Asche F, Hermansen Ø, Nystøyl R. Production cost and competitiveness in major salmon farming countries 2003–2018. Aquaculture. 2020;522:735089.

[77] Hersoug B. Why and how to regulate Norwegian salmon production? – The history of Maximum Allowable Biomass (MAB). Aquaculture. 2021;545:737144.

[78] Afewerki S, Asche F, Misund B, Thorvaldsen T, Tveteras R. Innovation in the Norwegian aquaculture industry. Rev Aquac. 2022;15:759–71. 10.1111/raq.12755.

[79] Scholaert F. Support for fishermen affected by the eastern Baltic cod closure | Think Tank | European Parliament. 2021.

[80] Polte P, Gröhsler T, Kotterba P, von Nordheim L, Moll D, Santos J, et al. Reduced reproductive success of Western Baltic Herring (Clupea harengus) as a response to warming winters. Front Mar Sci [Internet]. 2021 [cited 2025 Apr 10];8:589242. www.frontiersin.org

[81] Möllmann C, Cormon X, Funk S, Otto SA, Schmidt JO, Schwermer H, et al. Tipping point realized in cod fishery. Sci Rep [Internet]. 2021 [cited 2021 Aug 4];11:14259. https://www.nature.com/articles/s41598-021-93843-z 10.1038/s41598-021-93843-z PMC8275682 34253825

[82] von Storch H. Perceptions of an endangered Baltic Sea. Oceanologia. 2023;65:44–9.

[83] Cardinale M, Svedang H. Modelling recruitment and abundance of Atlantic cod, *Gadus morhua*, in the eastern Skagerrak-Kattegat (North Sea): evidence of severe depletion due to a prolonged period of high fishing pressure. Fish Res. 2004;69:263–82.

[84] Crain CM, Kroeker K, Halpern BS. Interactive and cumulative effects of multiple human stressors in marine systems. Ecol Lett. 2008;11:1304–15. 19046359 10.1111/j.1461-0248.2008.01253.x

[85] Liu J, Dietz T, Carpenter SR, Alberti M, Folke C, Moran E, et al. Complexity of coupled human and natural systems. Science. 1979;2007(317):1513–6. 10.1126/science.1144004 17872436

