# Supplementary material for: Identifying and addressing the anthropogenic drivers of global change in the North Sea: a systematic map
Source: Environ Evid. 2025 Dec 2;14:24. doi: 10.1186/s13750-025-00377-2 (PMC12673759; doi:10.1186/s13750-025-00377-2)
Supplement: Supplementary file 1 — Additional File 1: ROSES form, description: filled in ROSES form [file 13750_2025_377_MOESM1_ESM.pdf]

ROSES for Systematic Map Reports. Version 1.0

November 2017

Neal R Haddaway, Biljana Macura, Paul Whaley, and Andrew Pullin

Column descriptions

Section/sub-section - The sections or sub-sections listed below correspond to sections in CEE systematic review and map protocols and final reports: please see the relevant text under these subheadings for information within a specific review.

Topic - the section or sub-section is further broken down for the purposes of clarity.

Description/Further explanation - Details of what is needed in each section or sub-section are provided, along with practical advice and links to relevant guidance.

Checklist/Meta-data - Checklist items MUST be completed. Meta-data items correspond to information that should be reported as data or short descriptions that will be used to populate the Summary Record and Flow Diagram. Any meta-data items where stages were not performed (e.g. grey literature searching), this should be stated (e.g. 'Not performed').

Author response - authors should select or write a response for meta-data, or select 'yes' or 'no' for checklist items. Further details may be provided in Comments (see below).

Comments - authors may supplement their response with a brief comment.

Please cite as: Haddaway NR, Macura B, Whaley P, and Pullin AS. 2017. ROSES for Systematic Map Reports. Version 1.0. DOI: 10.6084/m9.figshare.5897299.

Item number	Section/sub-section	Topic	Description	Further explanation	Checklist/meta-data	Author response	Comments
1	Title	Title	The title must indicate that it is a systematic map, and should indicate if it	The title should normally be the same or very similar to the review	Meta-data	Identifying and addressing the anthropogenic drivers of global change in the North Sea: a systematic map.	
2	Type of review	Type of review	Select one of the following types of review: systematic map, systematic	See CEE Guidance on systematic mapping [1], and on amendments and	Meta-data	systematic map	
3	Authors' contacts	Authors' contacts	The full names, institutional addresses and email addresses for all authors		Checklist	Yes	
4	Abstract	Structured summary	The abstract of the manuscript must not exceed 500 words and must be		Checklist	Yes	
5	Background	Background	Describe the rationale for the review in the context of what is already	A theory of change and/or conceptual model should be presented that	Checklist	Yes	
6	Stakeholder engagement	Stakeholder engagement	The actual role of stakeholders throughout the review process (e.g. in		Checklist	Yes	Stakeholders other than researchers
7	Objective of the review	Objective	Describe the primary question and secondary questions (when	The primary question is the main question of the review. The secondary	Checklist	Yes	
8		Definition of the question	Provide reference to the question key elements, e.g. population(s),	For other question types see [4,5]	Meta-data	Population: any biotic and abiotic component of hte North Sea marine ecosystem (e.g., plant/animal species, seawater/sediment properties)	
9	Methods	Protocol	Provide citation, DOI or open-access link to published protocol.	The protocol should be peer-reviewed and publicly available online (open	Meta-data	https://doi.org/10.1186/s13750-021-00234-y	
10		Deviations from protocol	Describe any ways in which the final methods of the review deviate from		Checklist	Yes	
11	Searches	Search strategy	Detail the search strategy used, including: database names accessed,		Checklist	Yes	
12		Search string	Provide Boolean-style full search string and state the platform for which		Meta-data	TS=("climat*" NEA	Web of Science search syntax
13		Languages - bibliographic	List languages used in bibliographic database searches		Meta-data	English	
14		Languages – grey literature	List languages used in organisational website searches and web-based		Meta-data	English	
15		Bibliographic databases	Provide the number of bibliographic databases searched		Meta-data	4	Scopus, Web of Science, Pubmed, AquaDocs
16		Web-based search engines	Provide the number of web-based search engines searched		Meta-data	3	Google scholar, BASE, CORE
17		Organisational websites	Provide the number of organisational websites searched		Meta-data	7	WWF, UNEP-WCMC, IUCN, IPBES, CBD, OSPAR, ICES, EEA
18		Estimating comprehensiveness of	Describe the process by which the comprehensiveness of the search		Checklist	Yes	
19		Search update	Describe any update to searches undertaken during the conduct of the	Compulsory (if update performed). A search update is good practice if	Checklist	Yes	
20	Article screening and study	Screening strategy	Describe the methodology for screening articles/studies for relevance.		Checklist	Yes	
21		Inclusion criteria	Describe the inclusion criteria used to assess relevance of identified		Checklist	Yes	
22	Critical appraisal	Critical appraisal strategy	Describe here the method used for critical appraisal of study validity	Optional	Checklist	n/a	Critical appraisal not performed
23		Critical appraisal used in	Describe how the information from critical appraisal was used in	Compulsory if critical appraisal performed	Checklist	n/a	Critical appraisal not performed
24	Meta-data extraction and coding	Meta-data extraction and coding	Describe the method for meta-data extraction and coding for studies,		Checklist	Yes	
25		Approaches to missing data	Describe any process for obtaining and confirming missing or unclear		Checklist	Yes	
26	Data synthesis and presentation	Narrative synthesis strategy	Describe methods used for narratively synthesising the evidence base in		Checklist	Yes	
27		Knowledge gap and cluster	Describe the methods used to identify and/or prioritise key knowledge		Checklist	Yes	
28		Demonstrating procedural	Describe the role of systematic reviewers (who have also authored	Reviewers who have authored articles to be considered within the review	Checklist	Yes	
29	Results (review findings)	Description of review process	Describe the review process including the volume of evidence identified		Checklist	Yes	
30		Number of search results	Provide the number of search results from bibliographic databases	This number should not include web-based search engine or	Meta-data	32,622	
31		Number of search results after	Provide the total number of search results from bibliographic database	This number should not include web-based search engine or	Meta-data	22,511	
32		Full text screening excludes	Additional file containing list of and reasons for full text exclusions.		Checklist	Yes	
33		Title screening results	Provide the number of articles retained following title screening.	Optional if screening titles and abstracts together	Meta-data	n/a	
34		Abstract screening results	Provide the number of articles retained following abstract screening.	Optional if screening titles and abstracts together	Meta-data	n/a	
35		Title and abstract screening	Provide the number of articles retained following title and abstract	Optional if screening titles and abstracts separately	Meta-data	7,440	
36		Retrieval results	Provide the number of articles retrieved at full text.		Meta-data	5,795	
37		Unobtainable articles	Additional file containing list of unobtainable articles.		Checklist	Yes	
38		Full text screening results	Provide the number of articles retained following full text screening.		Meta-data	3,356	
39		Consistency checking: screening	Results of consistency checking at all stages (screening, meta-data		Checklist	Yes	
40		Narrative synthesis	Describe the body of evidence identified using figures and tables, avoiding		Checklist	Yes	
41		Systematic map database	Additional file containing meta-data and coding for included studies.		Checklist	Yes	
42		Limitations of the review	Discuss possible limitations in the methods used.		Checklist	Yes	
43		Limitations of the evidence base	Discuss possible limitations in the evidence base.		Checklist	Yes	
44	Conclusions	Knowledge gaps and clusters	Describe knowledge gaps (unrepresented or underrepresented subtopics		Checklist	Yes	
45		Implications for	Summarise the state of the evidence base and discuss the way in which	Reviews must not include practical environmental management	Checklist	Yes	
46		Implications for research	Discuss the way in which the identified evidence may inform research	In this section some advocacy for future research on the reviewed topic is	Checklist	Yes	
47	Declarations	Competing interests	Describe of any financial or non-financial competing interests that the		Checklist	Yes	

References

[1] James, K.L., Randall, N.P. and Haddaway, N.R., 2016. A methodology for systematic mapping in environmental sciences. Environmental Evidence, 5(1), p.7.

[2] Bayliss, H.R., Haddaway, N.R., Eales, J., Frampton, G.K. and James, K.L., 2016. Updating and amending systematic reviews and systematic maps in environmental management. Environmental Evidence, 5(1), p.20.

[3] Haddaway, N.R., Kohl, C., da Silva, N.R., Schiemann, J., Spök, A., Stewart, R., Sweet, J.B. and Wilhelm, R., 2017. A framework for stakeholder engagement during systematic reviews and maps in environmental management. *Environmental Evidence* , *6* (1), p.11.

[4] Collaboration for Environmental Evidence. 2018. Guidelines and Standards for Evidence synthesis in Environmental Management. Version 5.0. www.environmentalevidence.org/information-for-authors.

[5] Leeds Institute of Health Sciences. https://medhealth.leeds.ac.uk/info/639/information_specialists/1500/search_concept_tools. Accessed 12/11/2017.
